# TLR2/TLR4 activation induces Tregs and suppresses intestinal inflammation caused by *Fusobacterium nucleatum in vivo*

**DOI:** 10.1371/journal.pone.0186179

**Published:** 2017-10-09

**Authors:** Yin-ping Jia, Kun Wang, Zhu-jun Zhang, Ya-nan Tong, Dan Han, Chun-yu Hu, Qian Li, Yang Xiang, Xu-hu Mao, Bin Tang

**Affiliations:** 1 Department of Clinical Microbiology and Immunology, Southwest Hospital & College of Medical Laboratory Science, Third Military Medical University, Chongqing, China; 2 Emei Sanatorium of PLA Rocket Force, Emeishan, China; "INSERM", FRANCE

## Abstract

Toll-like receptors (TLRs) 2 and 4 play critical roles in intestinal inflammation caused by *Fusobacterium nucleatum* (*F*. *nucleatum*) infection, but the role of TLR2/TLR4 in regulation of proinflammatory cytokines remains unknown. In this study, through microarray analysis and qRT-PCR, we showed that TLR2/TLR4 are involved in the *F*. *nucleatum*-induced inflammatory signaling pathway in Caco-2 cells, C57BL/6 mice and human clinical specimens. In TLR2^-/-^ and TLR4^-/-^ mice, *F*. *nucleatum* infection resulted in increased colonization of the bacteria and production of the proinflammatory cytokines IL-8, IL-1β and TNF-α. In addition, the ratio of Foxp3^+^ CD4^+^ T cells in the total CD4^+^ T cells in TLR2^-/-^ and TLR4^-/-^ mice was less than that in wild-type mice, and the ratio in hybrid mice was more than that in knockout mice, which suggested that TLR2/TLR4 mediated the number of Tregs. Furthermore, it was observed that inflammatory cytokine levels were reduced in TLR2^-/-^ mice after Treg transfer. Thus, these data indicate that TLR2/TLR4 regulate *F*. *nucleatum*-induced inflammatory cytokines through Tregs in vivo.

## Introduction

*Fusobacterium nucleatum* (*F*. *nucleatum*), an anaerobic gram-negative bacterium, is normally prevalent in the oral cavity. This bacteria is the main cause of periodontal disease, and it is implicated in abscesses, inflammatory bowel disease (IBD), and colon cancer [1]. Intestinal inflammation is a well-known risk factor for colorectal cancer [2]. An accumulating number of investigations have found that *F*. *nucleatum* infection can lead to an inappropriate inflammatory response in intestinal epithelial cells [3], and *F*. *nucleatum* is significantly concentrated in inflammatory bowel tissue in patients with IBD [4–6]. However, the mechanism by which intestinal inflammation is induced by *F*. *nucleatum* infection remains unclear.

Toll-like receptors (TLRs) play a critical role in initiating both the innate and adaptive immune system defense [7–8]. TLRs are mainly expressed in the plasma membrane and the membrane of intracellular vesicles. Plasma membrane TLRs include TLR1, TLR2, TLR4, TLR5, and TLR6 [9]. Particularly, TLR2 is the signaling receptor for the gram-positive bacteria cell wall components peptidoglycan (PGN) and lipoteichoic acid (LTA), and TLR4 is the primary signaling receptor for the gram-negative bacterial cell wall component lipopolysaccharide. Se-Ra Park et al. reported that TLR2/TLR4 may take part in cytokine production by macrophages against *F*. *nucleatum* infection [10]. Accumulating studies have shown that *F*. *nucleatum* induces the epithelial inflammatory response through the nuclear factor-kappa B pathway [11]. These studies have focused primarily on periodontal epithelial cell models in *F*. *nucleatum* infection. However, studies of *F*. *nucleatum* infection in human intestinal epithelial cells and in vivo models are rare.

Regulatory T cells (Tregs) are a subpopulation of T cells that express the biomarkers CD4, Foxp3, and CD25 and are critical for mediating autoimmune disease and immune responses to infectious microorganisms [12]. During host immune responses, Tregs can be recruited and expanded to regulate the immune response [12]. To date, the mechanism underlying the immunomodulatory effect of Tregs by *F*. *nucleatum* infection remains largely unknown.

In the present study, the relationships between *F*. *nucleatum* and Toll-like receptors, Tregs and inflammation were investigated in Caco-2 cells and mice. We hypothesized that TLR2/TLR4 activation induces Tregs and suppresses intestinal inflammation caused by *Fusobacterium nucleatum* in mice.

## Materials and methods

### Ethics statement

The experiments involving specimens from human subjects (adult volunteers) and all animal experiments were approved by the Ethics Review Board of the Third Military Medical University (Chongqing; permit number 2011–04), and volunteers gave their written informed consent. Animal surgery was performed under sodium pentobarbital anesthesia, and CO_2_ inhalation was used to sacrifice animals at the indicated time points.

### Cell culture and bacterial strains

Caco-2 cells were obtained from the cell bank at the Chinese Academy of Sciences and were grown in DMEM (Gibco, 11965–092) containing 10% fetal bovine serum (Gibco, 10099–141) and 100 U/ml penicillin/streptomycin (Gibco, 15140–122) at 37°C in 5% CO_2_. *F*. *nucleatum* (ATCC 25586) was obtained from the American Type Culture Collection, and the specific culture method has been previously described [13].

### Mice and human clinical specimens

SPF female C57BL/6 mice (6 weeks old) and TLR2^-/-^ and TLR4^-/-^ mice were obtained from the experimental animal center of the Third Military Medical University (Chongqing, China). TLR2^-/+^ and TLR4^-/+^mice were obtained by crossing TLR2^-/-^ and TLR4^-/-^mice with WT mice. Mice were fed with *F*. *nucleatum* (10^8^ CFU per day) for a period of 1 week. Sham treatment consisted of TSB (tryptic soy broth, BD). After 2 months, all the mice were sacrificed. The intestinal tissues were rapidly immersed in liquid nitrogen for qRT-PCR and prepared with paraformaldehyde for H.E staining, and splenocytes from the mice were prepared for flow cytometry analysis.

Patients who underwent colonoscopy at Southwest Hospital, Chongqing, China during January-December 2016 were recruited. In total, 19 (10 women and 9 men, with a mean age of 41±10 years) patients were eligible for enrollment into the *F*. *nucleatum*-positive group on the basis of their 16s rRNA gene copy number and a positive *F*. *nucleatum* culture. In addition, 6 (3 women and 3 men with a mean age of 30±5 years) with normal intestinal mucosa were eligible for enrollment into the *F*. *nucleatum*-negative group. The intestinal specimens were derived from the descending colon of *F*. *nucleatum*-positive patients and obtained during endoscopy. Three biopsies were taken from the colon for RNA extraction, *F*. *nucleatum* culture, and histological examination. Histological examination was performed by two pathologists according to the updated Sydney classification [14].

### RNA preparation and gene expression analysis using qPCR

When the tissues were removed from the liquid nitrogen tank, liquid nitrogen was added to a ceramic grinder, and the tissues were quickly ground into powder. Then, 1 ml of TRIzol® Reagent was used to collect the powder in a 1.5 ml-tube. Total RNA of cells or tissues was extracted using TRIzol® Reagent (Life Technologies), and cDNA was generated using a PimeScript TMRT Reagent Kit (Takara). Real-time quantitative RT-PCR was performed on a Bio-Rad IQ5 instrument (Bio-Rad Laboratories, Inc.) to investigate the RNA expression level of Toll-like receptors, *F*. *nucleatum* 16s rRNA gene copies and proinflammatory cytokines in mice or cells, and performed using the following parameters: 95°C for 2 min followed by 40 cycles of 95°C for 15 sec and 60°C for 30 sec. The mRNA level of ACTB was used as an internal control. The primer sequences are given in S1 Table.

### ELISA assays

C57BL/6 wild-type, hybrid, TLR2^-/-^ and TLR4^-/-^ mice (n = 8 in each group) were infected with *F*. *nucleatum* (10^8^ CFU per day) for 1 week. After 2 months, the blood serum of the mice was used to determine IL-1β, IL-8 and TNF-α levels using enzyme-linked immunosorbent assay (ELISA) kits (Boster Biotechnology Co., Ltd., Wuhan, China) according to the manufacturer’s instructions.

### Microarray analysis

Caco-2 cells were infected with *F*. *nucleatum* (MOI = 100:1) or not (control) for 24 hours. Total RNA of cells was extracted using TRIzol® Reagent (Life Technologies), and cDNA was generated using a PimeScript TMRT Reagent Kit (Takara). Affymetrix GeneChip® Human Transcriptome Array 2.0 chips were used to analyze 10 specimens (5 with *F*. *nucleatum* infection and 5 control), which were uploaded to GEO with the accession number GSE102573. Probe cell intensity data (CEL) data from the human transcriptome arrays was analyzed using Expression Console Software. The RMA analysis was used to create CHP files. Further statistical analysis was performed with Affymetrix® Transcriptome Analysis Console (TAC) Software to obtain differentially expressed genes and different gene signaling pathways.

### Flow cytometry

The monoclonal antibodies anti-CD3-PE, anti-CD4-APC, and anti-CD25-FITC and all isotype controls were purchased from BD Biosciences-Pharmingen, and anti-mFoxp3-PE was obtained from eBioscience. We used FACSCalibur (BD) and CELLQuest software (version 3.3; BD Biosciences-Pharmingen) to analyze lymphocyte surface markers. Briefly, 50–100 μl of blood was collected from mice in heparin-coated tubes, and erythrocytes were lysed using standard protocols. The remaining lymphocytes were washed three times with PBS and incubated with anti-mFoxp3-PE and anti-CD4-APCs and subsequently analyzed on a FACSCalibur (BD) flow cytometer with CELLQuest software (version 3.3; BD Biosciences-Pharmingen) as previously published [15].

### In vivo Treg proliferation

Lymphocytes were isolated from spleens of C57BL/6 mice. We used anti-mFoxp3-PE to stain the cells and anti-PE MACS beads (Miltenyi Biotec) to isolate them. Contaminating CD8^+^ T cells and B cells were removed via negative depletion using Dynal beads. Pure (up to 95%) Foxp3^+^CD4^+^ T cells (Tregs) were labeled with CFSE (5 μM), a new dye for fluorescent labeling of living cells, and injected i.p. into TLR2^–/–^mice (2 × 10^6^ per mouse). After four hours, the mice were challenged with *F*. *nucleatum* (10^8^ CFU per day) for a period of 1 week. The splenocytes were stained with anti-Foxp3-PE and anti-CD4-APCs to confirm that the CFSE^+^ cells were still Foxp3^+^. The production of proinflammatory cytokines in the blood of the mice was measured.

### Statistical analysis

The significance of differences between groups was assessed with one-way ANOVA, and analysis of *F*. *nucleatum* infection vs. control was performed with a t-test. Correlation analysis was performed using Spearman’s correlation test. All statistics were performed using SPSS Statistics 19 software. A *P* value of less than 0.05 was considered significant and is denoted as follows: * *P*<0.05; ** *P*<0.01.

## Results

### TLR2/TLR4 are involved in the *F*. *nucleatum*-induced inflammatory signal pathway

**Fig 1:**
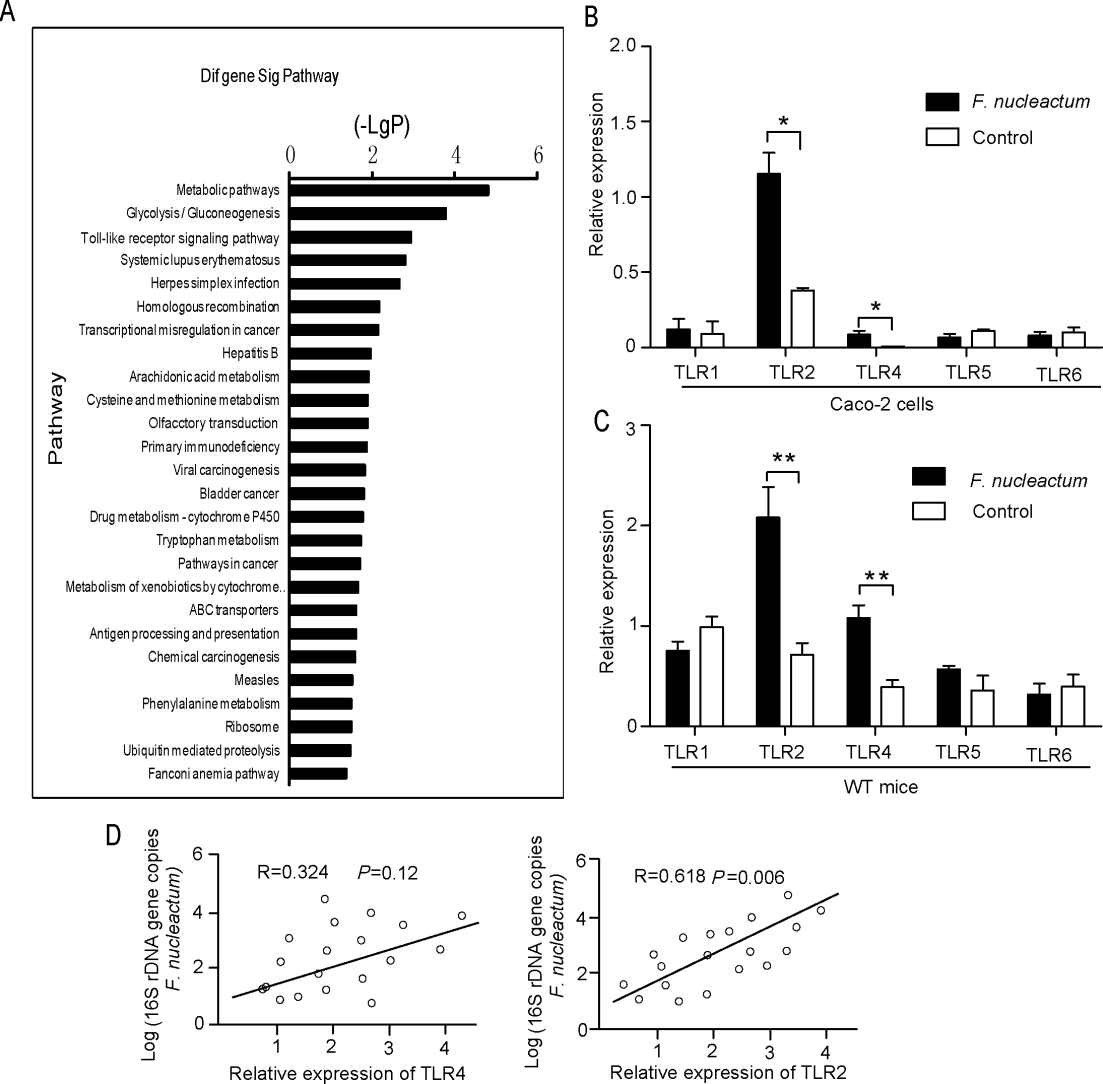
TLR2/TLR4 are involved in the *F*. *nucleatum*-induced inflammatory signaling pathway. (A) Signaling pathways of differentially expressed genes in Caco-2 cells infected with *F*. *nucleatum*. Pathway analysis was predominantly based on the KEGG database. An AP-value of <0.05 and an FDR of <0.05 in the two-sided Fisher’s exact test were considered statistically significant. The vertical axis represents the pathway category, and the horizontal axis represents the log 10 (*P* value) of these significant pathways. (B and C) Caco-2 cells or C57BL/6 mice were infected with *F*. *nucleatum* for 24 h or 1 week. The mRNA levels of TLR1, TLR2, TLR4, TLR5 and TLR6 were determined by qRT-PCR. (D) The correlation between TLR2/TLR4 and *F*. *nucleatum* 16s rRNA gene copies in human clinical specimens was examined by Spearman correlation analysis and found to be positive (TLR4/*F*. *nucleatum*, R = 0.324, *P* = 0.12; TLR2/*F*. *nucleatum*, R = 0.618, *P* = 0.006).

Recently, we reported that *F*. *nucleatum* infection could induce production of proinflammatory cytokines through reactive oxygen species (ROS) in Caco-2 cells [13]. In the course of the study, via an Affymetrix microarray analysis in Caco-2 cells infected with *F*. *nucleatum*, we observed that the Toll-like receptor signaling pathway may participate in *F*. *nucleatum-*induced inflammation (Fig 1A). To further confirm whether *F*. *nucleatum* induces expression of TLRs, the RNA expression of TLRs was investigated in Caco-2 cells or C57BL/6 mice using real-time PCR. There was an increase in the RNA expression of TLR2 and TLR4 in cells or mice infected with *F*. *nucleatum* compared to controls, while *F*. *nucleatum* infection had no significant effect on the RNA expression of TLR1, TLR5 and TLR6 (Fig 1B and 1C), which suggested that TLR2 and TLR4 may be involved in *F*. *nucleatum-*induced inflammation. To confirm that the TLR2 and TLR4 expression was a result of *F*. *nucleatum* infection, Spearman correlation analysis was used to compare the relative expression levels of TLR2/TLR4 and 16s rRNA gene copies of *F*. *nucleatum* in human clinical specimens. Two statistically significant positive correlations from a total of 19 *F*. *nucleatum*-positive intestinal tissues (TLR4/*F*. *nucleatum*, R = 0.324, *P* = 0.12; TLR2/*F*. *nucleatum*, R = 0.618, *P* = 0.006) (Fig 1D) were observed, and these results indicated that TLR2 expression is positively correlated with *F*. *nucleatum* infection in human samples.

### TLR2/TLR4 signaling modulates *F*. *nucleatum*-induced inflammation in vivo

**Fig 2:**
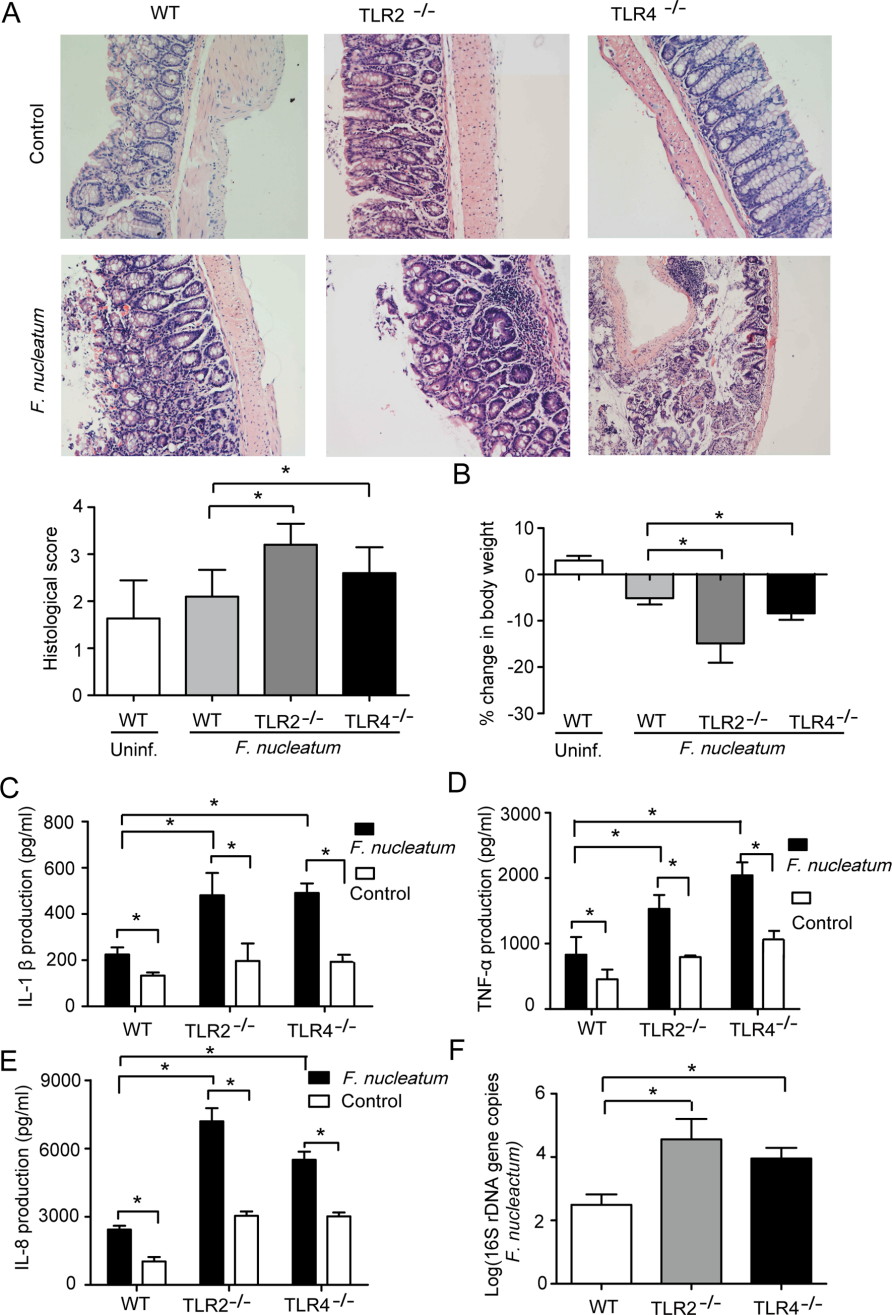
TLR2/TLR4 signaling modulates *F*. *nucleatum*-induced inflammation in vivo. (A) Histological inflammation scores (H&E staining) of the gastric mucosa of C57BL/6 wild-type mice and TLR2^-/-^ and TLR4^-/-^ mice with *F*. *nucleatum* infection for 1 week. The intensity of staining is shown in the right graph, and the data are expressed as the mean±SEM. (B) The body weight of C57BL/6 wild-type mice and TLR2^-/-^ and TLR4^-/-^ mice after *F*. *nucleatum* infection. (C, D and E) The production of IL-8, IL-1β and TNF-α in C57BL/6 wild mice and TLR2^-/-^ and TLR4^-/-^ mice infected with *F*. *nucleatum* for 1 week, as assessed by enzyme-linked immunosorbent assay (ELISA). (F) The number of *F*. *nucleatum* 16s rRNA gene copies in the gastric mucosa of C57BL/6 wild mice and TLR2^-/-^ and TLR4^-/-^ mice based on qRT-PCR.

To further determine the role of TLR2/TLR4 in *F*. *nucleatum*-induced inflammation, we first examined the intestinal inflammation level in C57BL/6 wild-type mice and TLR2^-/-^ and TLR4^-/-^ mice infused with the bacteria. As shown in Fig 2A, *F*. *nucleatum* significantly increased the level of intestinal inflammation in TLR2^-/-^ and TLR4^-/-^ mice compared to that in the control C57BL/6 mice. We also observed that the body weight of TLR2^-/-^ and TLR4^-/-^ mice was less than that of wild-type mice (Fig 2B). In addition, the protein levels of proinflammatory cytokines (IL-8, IL-1β and TNF-α) were also examined by ELISA in C57BL/6 wild-type mice and TLR2^-/-^ and TLR4^-/-^ mice. As shown in Fig 2C, 2D and 2E, the levels of IL-8, IL-1β and TNF-α were significantly higher in TLR2^-/-^ and TLR4^-/-^ mice infected with *F*. *nucleatum* compared with wild-type mice. To further confirm that the TLR2 and TLR4 expression was a result of *F*. *nucleatum* infection, we detected *F*. *nucleatum* 16s rRNA gene copies in C57BL/6 wild-type mice and TLR2^-/-^ and TLR4^-/-^ mice. There was a higher number of *F*. *nucleatum* gene copies in the two knockout mice than in the wild-type mice (Fig 2F). These results suggested that TLR2/TLR4 signaling inhibited *F*. *nucleatum*-induced inflammation in vivo.

### *F*. *nucleatum* infection increased the ratio of regulatory T cells in vivo

**Fig 3:**
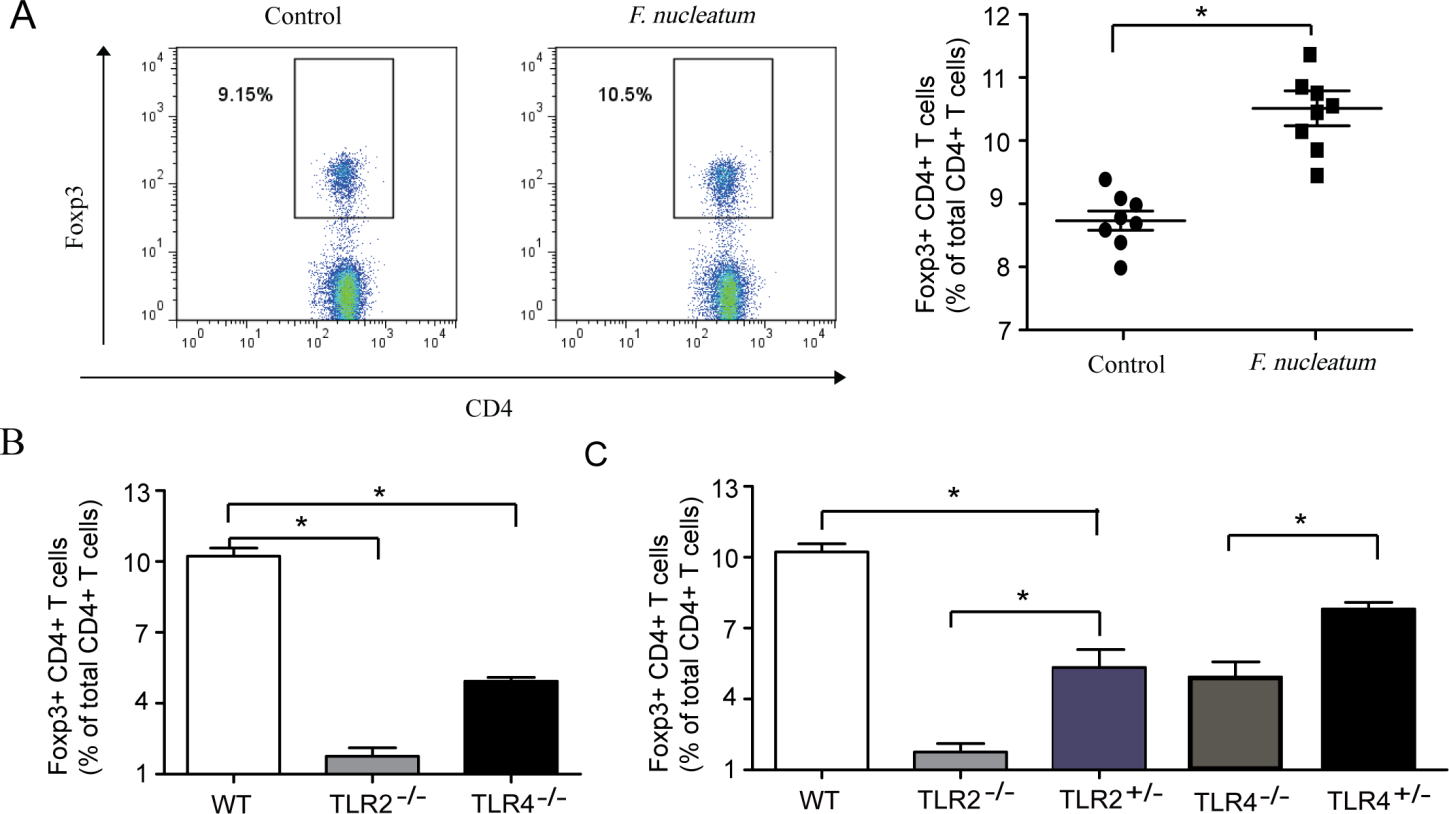
*F*. *nucleatum* infection increased the ratio of regulatory T cells in vivo. (A) Sixteen C57BL/6 wild mice were divided into two groups (n = 8). After eight C57BL/6 wild-type mice were infected with *F*. *nucleatum* for 1 week, FoxP3 and CD4 expression was analyzed with FACS after fixation and permeabilization, and a gating control was used. The *P* value (Mann–Whitney) is indicated. (B) Detection of Foxp3^+^ CD4^+^ T cells in total CD4^+^ T cells in wild-type, TLR2^-/-^ and TLR4^-/-^ mice. (C) Detection of Foxp3^+^ CD4^+^ T cells in total CD4^+^ T cells in wild-type, hybrid, TLR2^-/-^ and TLR4^-/-^ mice.

As we know, TLR2/TLR4 induces the expression of proinflammatory cytokines via NF-κB. However, in our study, TLR2/TLR4 signaling inhibited *F*. *nucleatum*-induced inflammation in vivo. We speculated that there are some negative regulation mechanisms in *F*. *nucleatum*-induced inflammation in vivo. To clarify this problem, we detected the regulatory T cells (Tregs), which have the ability to suppress inflammation in several diseases, in the *F*. *nucleatum* infection in vivo model [12, 16–17]. As shown in Fig 3A, *F*. *nucleatum* infection increased the proportion of Foxp3^+^ CD4^+^ T cells in total CD4^+^ T cells. To determine the role of TLR2/TLR4 in regulating Tregs, we first examined the number of Foxp3^+^ CD4^+^ T cells after *F*. *nucleatum* infection in C57BL/6 wild-type mice and in TLR2^-/-^ and TLR4^-/-^ mice. The ratio of Foxp3^+^ CD4^+^ T cells in total CD4^+^ T cells in TLR2^-/-^ and TLR4^-/-^ mice was less than that in wild-type mice (Fig 3B). The results suggested that TLR2/TLR4 can regulate Tregs, which is consistent with a previous study [18]. In addition, we hybridized wild-type mice with knockout mice to obtain TLR2^+/-^ and TLR4^+/-^ mice, which were also infected with *F*. *nucleatum*. It was observed that the ratio of Foxp3^+^ CD4^+^ T cells in TLR2^+/-^ and TLR4^+/-^ mice was more than that in TLR2^-/-^ and TLR4^-/-^ mice and less than that in wild-type mice (Fig 3C). These data indicate that TLR2/TLR4 can modulate the number of Tregs.

### TLR2/TLR4 regulate *F*. *nucleatum*-induced inflammatory cytokines through Tregs in vivo

**Fig 4:**
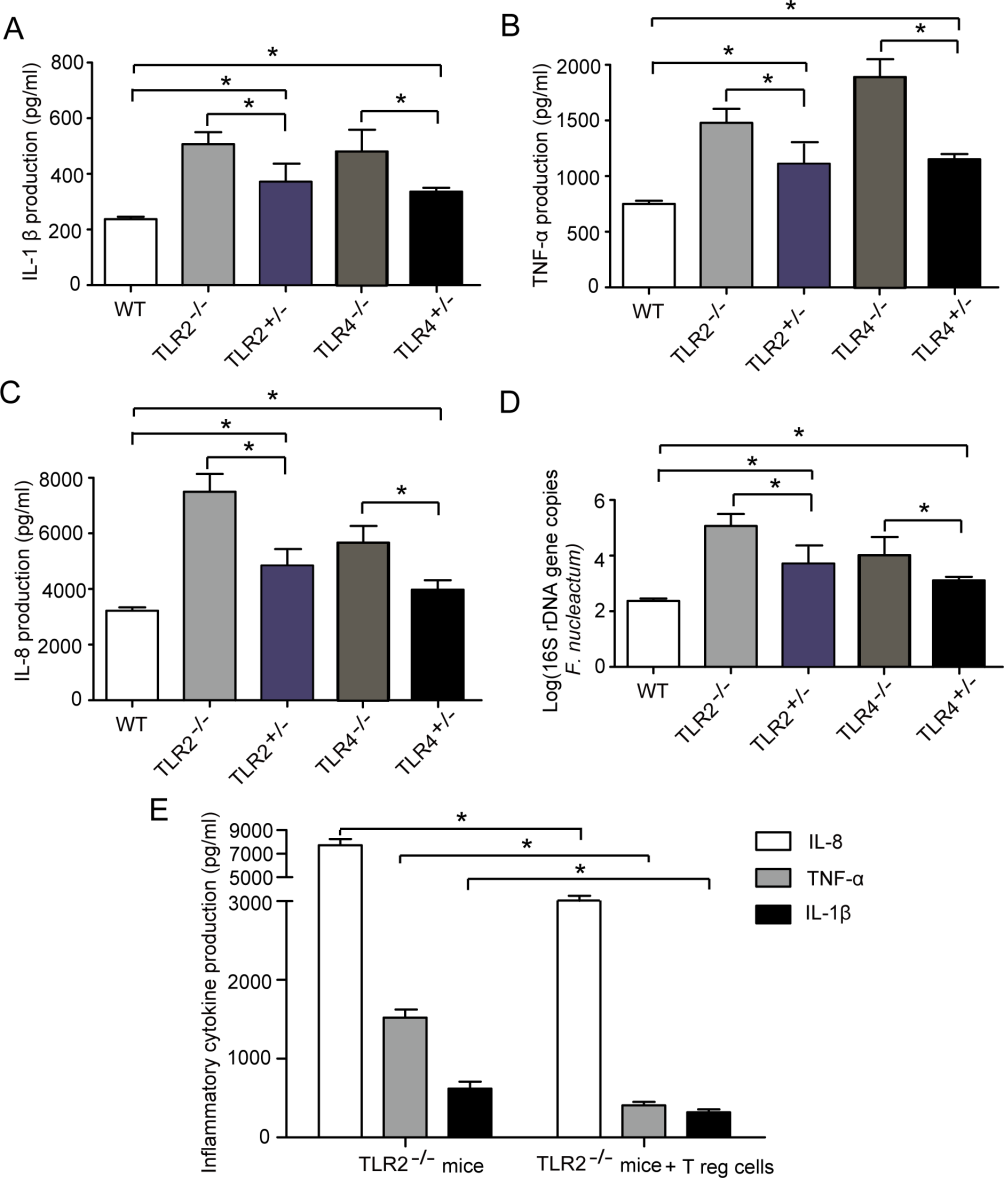
TLR2/TLR4 regulate *F*. *nucleatum*-induced inflammatory cytokines through Tregs in vivo. (A, B and C) Production of IL-8, IL-1β and TNF-α in C57BL/6 wild-type mice and in hybrid, TLR2^-/-^ and TLR4^-/-^ mice infected with *F*. *nucleatum* for 1 week, as assessed by enzyme-linked immunosorbent assay (ELISA). (D) The number of *F*. *nucleatum* 16s rRNA gene copies in the gastric mucosa of C57BL/6 wild-type mice and in hybrid, TLR2^-/-^ and TLR4^-/-^ mice determined by qRT-PCR. (E) After transfer of Tregs from wild-type mice to TLR2^-/-^ mice, the production of IL-8, IL-1β and TNF-α in TLR2^-/-^ mice infected with *F*. *nucleatum* for 1 week and in the uninfected group was assessed by ELISA.

To further confirm that TLR2/TLR4 regulate *F*. *nucleatum*-induced inflammatory cytokines in vivo, we examined inflammatory cytokine levels in the hybrid and knockout mice. As shown in Fig 4A, 4B and 4C, the levels of IL-8, IL-1β and TNF-α were significantly higher in TLR2^-/-^ and TLR4^-/-^ mice infected with *F*. *nucleatum* than in TLR2^+/-^ and TLR4^+/-^ mice. In addition, the number of *F*. *nucleatum* 16s rRNA gene copies in TLR2^-/-^ and TLR4^-/-^ mice was higher than that in TLR2^+/-^ and TLR4^+/-^ mice (Fig 4D). To further clarify whether the Treg subset is involved in *F*. *nucleatum*-induced inflammatory cytokine production in vivo, we transferred Tregs from wild-type mice to knockout mice. It was observed that the inflammatory cytokine levels were reduced in TLR2^-/-^ mice after transfer of Tregs (Fig 4E). These data indicate that TLR2/TLR4 regulate *F*. *nucleatum*-induced inflammatory cytokines through Tregs in vivo.

## Discussion

In our previous study, we reported that *Fusobacterium nucleatum*-induced impairment of autophagic flux enhances the expression of proinflammatory cytokines via ROS in Caco-2 cells [13]. Many studies have also reported that *F*. *nucleatum* induced proinflammatory cytokines through different pathways [19–20]. However, in the present study, it is first proposed that TLR2/TLR4 regulate *F*. *nucleatum*-induced inflammatory cytokines through Tregs in vivo. This novel model is supported by the following data: i) TLR2/TLR4 are involved in the *F*. *nucleatum*-induced inflammatory signaling pathway, ii) TLR2/TLR4 signaling modulates *F*. *nucleatum*-induced inflammation in vivo, iii) *F*. *nucleatum* infection increased the ratio of regulatory T cells in vivo, and iv) TLR2/TLR4 regulate *F*. *nucleatum*-induced inflammatory cytokines through Tregs in vivo.

Many studies have reported that Toll-like receptors participate in immune regulation in various in vitro and in vivo models, but the specific mechanism is still unclear. In studies of *F*. *nucleatum*-induced immune responses, there have been inconsistent results regarding the involvement of TLR2 and TLR4. In peritoneal macrophages from TLR2-deficient or TLR4-mutant (C3H/H3J) mice, the production of TNF-α and reactive oxygen species (ROS) was significantly lower than that in control cells from WT mice with *F*. *nucleatum* infection [21–22]. After treatment with TLR2 and TLR4 neutralizing antibodies in human periodontal ligament cells, *F*. *nucleatum*-mediated production of cytokines and chemokines was decreased significantly [23]. In addition, IL-8 production induced by *F*. *nucleatum* was increased in HEK cells transfected with CD14-TLR2 but not in cells transfected with CD14-TLR4 [24]. The present study is the first to demonstrate that *F*. *nucleatum* induces increased production of IL-8, IL-1β and TNF-α in TLR2^-/-^ and TLR4^-/-^ mice compared with wild-type mice. Nonetheless, it is well known that TLR2/4 signaling is capable of promoting inflammation through the NF-κB or AP-1 pathway. These discrepancies seem to be due to in vitro/in vivo model differences. It is difficult to assess the role of TLR2/4 in immune protection or pathogenesis of infection in an in vitro model because inflammation is a very complicated process that involves several types of immune cells and cytokines.

Tregs are emerging as major regulators of our immune systems, and dysfunctional Tregs can lead to various immune diseases. Effective immune responses can be hindered by Treg-mediated suppression, and the immune responses are crucial for elimination of tumors and infections. Therefore, Tregs are a critical cell subset in maintaining immune balance. This study is the first to report that TLR2 triggered by *F*. *nucleatum* infection can modulate Treg function. As shown in Fig 3B, the ratio of Foxp3^+^ CD4^+^ T cells in the total CD4^+^ T cells in TLR2^-/-^ and TLR4^-/-^ mice was less than that in wild-type mice. The results indicate that TLR2 and TLR4 can modulate the number of Tregs, which is consistent with the results of other studies [18]. Roger P.M. Sutmuller et al. observed that Treg numbers were decreased in TLR2-deficient mice compared with C57BL/6 wild-type mice [25]. In addition, we crossed wild-type mice with knockout mice to obtain TLR2^+/-^ and TLR4^+/-^ mice. After *F*. *nucleatum* infection, the ratio of Foxp3^+^ CD4^+^ T cells in TLR2^+/-^ and TLR4^+/-^ mice was more than that in TLR2^-/-^ and TLR4^-/-^ mice and less than that in wild-type mice (Fig 3C). These data further indicate that TLR2/TLR4 can modulate the number of Tregs.

To sum up, the present study has major implications for our understanding of intestinal inflammation regulated by both Tregs and TLR2/TLR4 in *F*. *nucleatum* infection. Although further experiments are needed to determine the specific mechanism of Treg and TLR2/TLR4 regulation in *F*. *nucleatum* infection, our studies establish a basis to evaluate the role of Tregs and TLR2/TLR4 during *F*. *nucleatum* infection. Ultimately, the knowledge that TLR2/TLR4 can be used to expand and modulate Tregs will lead to new methods to treat *F*. *nucleatum* infection.

## Supporting information

S1 Table The primer sequences in the manuscript. (DOCX)
