# Supplementary material for: TLR2/TLR4 activation induces Tregs and suppresses intestinal inflammation caused by Fusobacterium nucleatum in vivo
Source: PLoS One. 2017 Oct 9;12(10):e0186179. doi: 10.1371/journal.pone.0186179 (PMC5633168; doi:10.1371/journal.pone.0186179)
Supplement: S1 Table — (DOCX) [file pone.0186179.s001.docx]

MATERIAL & METHODOLOGY

Table 1. Sequences of primers used in the study

| Gene Symbol | Primer | Primer Sequence (5′ → 3′) | Lengths of product(bp) |
| --- | --- | --- | --- |
| Human-TLR1 | F | CCAAATGGAACAGACAAGCAGG | 116 |
|  | R | ATGAAGACCCTGGCCACAAA |  |
| Human-TLR2 | F | GGTTCAAGCCCCTTTCTTCT | 117 |
|  | R | TTCCCACTCTCAGGATTTGC |  |
| Human-TLR4 | F | CCAGGATGAGGACTGGGTAA | 152 |
|  | R | CCTTTCGGCTTTTATGGAAA |  |
| Human-TLR5 | F | TTTTGCCACAGAAACAATGC | 146 |
|  | R | TGGAGAAGCCGAAGGTAAGA |  |
| Human-TLR6 | F | AGAGGAAGCCCACTAAAGGAC | 104 |
|  | R | ACAGTCACAGCCAACACCAG |  |
| Human-ACTB | F | TTCCTTCCTGGGCATGGAGTCC | 145 |
|  | R | TGGCGTACAGGTCTTTGCGG |  |
| Mouse-TLR1 | F | TAAAGAGGCCAAACGCAAAC | 140 |
|  | R | TATCAGGACCCTCAGCTTGG |  |
| Mouse-TLR2 | F | CCACTGCCCGTAGATGAAGT | 153 |
|  | R | CCCATTGAGGGTACAGTCGT |  |
| Mouse-TLR4 | F | AGGTTGAGAAGTCCCTGCTG | 146 |
|  | R | CGAGGCTTTTCCATCCAATA |  |
| Mouse-TLR5 | F | CTCAAACACCTGGATGCTCA | 137 |
|  | R | AGACACACCGTCTTCCTGCT |  |
| Mouse-TLR6 | F | CTGCAAAGTTCCGACAACTG | 137 |
|  | R | ATGGAGAACGGTGGTATTGG |  |
| Mouse-ACTB | F | GACTTTGTCTGTTGTTTTG | 187 |
|  | R | TGCACTAATATTGGTCTCA |  |
| 16s rRNA of *F. nucleatum* | F | TGGCATAGCTTCACCTTTGA | 144 |
|  | R | CAAAGACTTGGGGAAATGGA |  |
|  |  |  |  |
